# Efficacy of PIVKA-II in prediction and early detection of hepatocellular carcinoma: a nested case-control study in Chinese patients

**DOI:** 10.1038/srep35050

**Published:** 2016-10-12

**Authors:** Rentao Yu, Xiaomei Xiang, Zhaoxia Tan, Yi Zhou, Haoliang Wang, Guohong Deng

**Affiliations:** 1 Department of Infectious Diseases, Southwest Hospital, the Third Military Medical University, Chongqing, China; 2 Chongqing Key Laboratory of Infectious Diseases, Southwest Hospital, Third Military Medical University, Chongqing, China; 3 Institute of Immunology, Third Military Medical University, Chongqing, China

## Abstract

Prognosis of hepatocellular carcinoma (HCC) remains unsatisfying due to a lack of early detecting methods. Protein Induced by Vitamin K Absence or Antagonist-II (PIVKA-II) has been proved to be an efficient biomarker for HCC. However, the predicting efficacy of PIVKA-II has barely been reported. In the Hepatitis Biobank of Southwest Hospital (HBS) cohort at Southwest Hospital, we did a two-stage nested case-control study. Totally, 45 HCC cases versus 138 matched controls were enrolled to compare levels of α-fetoprotein (AFP) and PIVKA-II in sequential sera at −12, −9, −6, −3 and 0 months before imaging diagnosis. Levels of both PIVKA-II and AFP in HCC cases elevated significantly at all time points compared with controls. In validation stage, the sensitivity and specificity of PIVKA-II at baseline were 58.3% and 92.6%, and AFP were 75.0% and 91.7%. AFP-/PIVKA-II+ patients covered 27.4%, 29.4% and 19.6% at M-12, M-6 and M-0, respectively, while AFP+/PIVKA-II- patients covered 25.5%, 19.6% and 17.7%, respectively. Both PIVKA-II and AFP have the potential for HCC prediction, while PIVKA-II has a better positive rate than AFP before diagnosis.

Hepatocellular carcinoma (HCC) ranks the sixth most common malignancy worldwide and accounts for about 5.6% of all human cancers^1^,^2^. Despite encouraging progress in diagnosis and treatment of HCC, the prognosis remains unsatisfying with 5-year overall survival rate lower than 10%^3^, however, the rate will reach 50–74% if an early detection and intervention is properly carried out^4^. But disappointingly, about 50% HCC cases diagnosed are at advanced stage when curative treatments are limited^5^. Etiologically, chronic viral hepatitis is the most frequent risk factors for HCC, of which 60% cases are attributed to hepatitis B in East Asia^6^. Therefore, a feasible surveillance strategy for at-risk populations is necessary.

Current guidelines of each organization provide some practicable surveillance plans. EASL and AASLD recommends hepatic ultrasound every 6 months^6^,^7^, and APASL adds α-fetoprotein (AFP) besides ultrasound^8^. Yet, it has been reported that neither ultrasound nor AFP is reliable, with a sensitivity of 63% for ultrasound alone, and there is no additional benefit if AFP is complimented^9^. Consequently, biomarkers for early detection of HCC are highly required.

Protein Induced by Vitamin K Absence or Antagonist-II (PIVKA-II), also known as Des-γ-carboxy-prothrombin (DCP), is another marker specific for HCC from 1984^10^. An elevated serum level of PIVKA-II is reported to be associated with HCC. Many studies have shown that PIVKA-II is applicable for HCC surveillance and has been written into the guideline of JSH^11^, which achieves remarkably good results^5^.

To the best of our knowledge, most studies on HCC biomarkers have concentrated on the diagnostic accuracy of HCC or its predictive role in carcinoma progression^12^,^13^. But few have focused on at-risk cohorts to evaluate these markers in predict HCC before clinical diagnosis. An early study from HALT-C trail showed that neither PIVKA-II nor AFP is optimal for early detection of HCC^14^, but it was conducted on hepatitis C virus (HCV)-based HCC patients. Considering different mechanisms of hepatocarcinogenesis among different aetiologies^15^, the roles of PIVKA-II in hepatitis B virus (HBV)-associated HCC might vary a lot.

We did a nested case-control study, using retrospectively collected sera from patients with HCC and at-risk controls to identify if PIVKA-II could identify preclinical HCC.

## Results

### Discovery stage

The clinical characteristics at baseline point were shown in Table 1. The female ratio for HCC patients and controls was 20.0% and the median age were 48.0 (40.0–66.0) versus 47.0 (39.0–67.0). HBV DNA were controlled under detecting level in most patients and controls (67.6% vs 73.3%), but cirrhotic basis was 66.7%, and 73.3% were diagnosed at BCLC 0 or A stage. AST, ALT, ALP, TBIL and TBA showed no differences between patients and controls, but TP level elevated significantly in HCC patients compared with CHB controls (P = 0.024).

**Figure 1:**
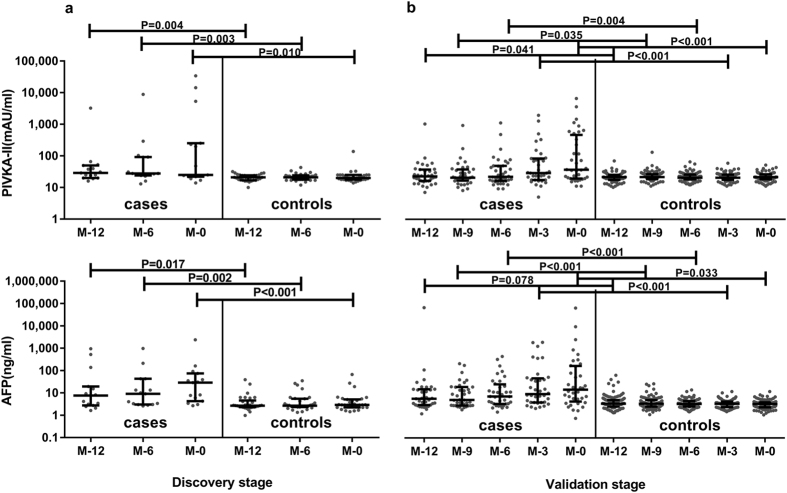
Levels of PIVKA-II and AFP at all time points at discovery stage and validation stage. Gray points refer to each value and dark lines refer to the 25th and 75th percentile values with a long dark line indicating median levels. P values were calculated by Mann-Whitney tests between two columns.

For HCC biomarkers, Fig. 1 shows that levels of both PIVKA-II and AFP in HCC cases were significantly higher than controls at all time points. PIVKA-II values decreased a little from 29.0 (20.0–50.0) at M-12 to 25.0 (22.0–253.0) mAU/ml at M-0 in the HCC cases (P = 0.678), and from 21.0 (17.0–25.0) at M-12 to 20.0 (18.0–25.0) mAU/ml at M-0 in the controls (P = 0.836). However, AFP levels elevated a little at M-0 compared with M-12 in both HCC cases and controls, but there was no statistical significance: from 7.6 (2.8–19.6) to 28.9 (4.3–74.6) ng/ml in HCC cases (P = 0.199), from 2.7 (2.3–4.6) to 2.9 (2.3–5.1) ng/ml in controls (P = 0.478).

ROC curve was drawn and AUC was calculated at diagnostic time to provide best cut-off values for validation stage analysis. The AUC for AFP was 0.853 (0.738–0.969) and for PIVKA-II was 0.739 (0.568–0.910). Based on ROC curve, 7.1 ng/ml (Youden Index = 0.567) and 40.5 mAU/ml (Youden Index = 0.433) were calculated as proper cut-off values.

To clarify if cirrhosis basis and liver function could affect the levels of two biomarkers, logistic regression was performed to evaluate the association between each parameter and two biomarkers. The results showed that only PIVKA-II (OR = 11.023, P = 0.022) and AFP (OR = 11.086, P = 0.019) were risk factors for the state of HCC. Other factors like liver functions were not confounding factors in this research (see Table 1, Supplemental Content, which illustrates Multivariable analysis of HCC in discovery stage). Based on this results, matching terms in validation stage were still gender, age and cirrhotic basis.

### Validation stage

Baseline information of HCC patients and controls was shown in Table 1. The female ratio for HCC patients and controls was 27.8% and the median age were 45.5 (39.5–52.0) versus 46.0 (39.0–52.0). In HCC patients, half were diagnosed at early stage and copy number of HBV DNA in 80.5% patients (79.6% in controls) was undetectable, but 75% got cirrhotic basis. For laboratory indexes, TP, ALP, TBIL and TBA levels elevated significantly in HCC patients compared with CHB controls, but AST and ALT showed no difference between patients and controls (P = 0.585 and.807, respectively).

In validation stage, sera at M-12, M-9, M-6, M-3 and M-0 were tested of AFP and PIVKA-II levels during more than 3-year follow-ups. Similar to discovery stage, levels of both PIVKA-II and AFP in HCC cases elevated significantly compared with controls at all time points (Fig. 1). PIVKA-II values elevated during the one-year follow-up from 22.0 (16.0–36.0) to 35.5 (19.0–437.5) mAU/ml (P = 0.008) and AFP values from 5.2 (2.8–13.6) to 13.1 (3.9–143.0) ng/ml (P = 0.024) but remained unchanged in the controls: 21.0 (17.0–25.0) and 21.0 (18.0–26.0) mAU/ml for PIVKA-II, 3.2 (2.4–4.6) and 3.1 (2.2–3.6) ng/ml for AFP, respectively.

**Figure 2:**
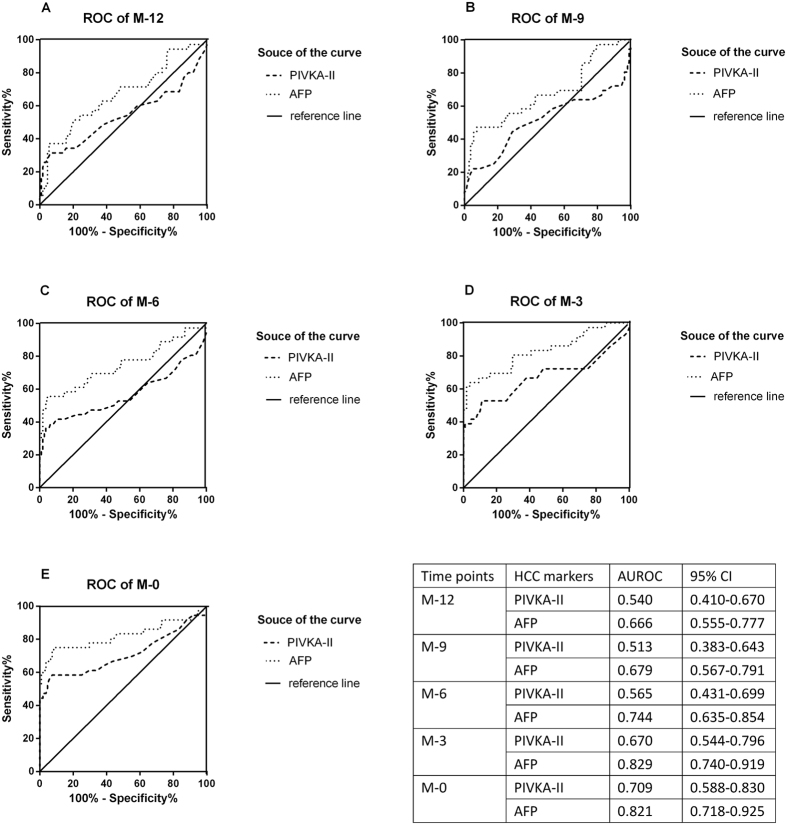
ROC curves at all time points at validation stage. The area under the curve is shown with its 95% confidence intervals. At each point, the AUC of AFP is better than PIVKA-II.

Figure 2 shows that AFP had a better predictive and diagnostic performance than PIVKA-II at all time points compared with CHB controls according to ROC curve. The AUC for each time were 0.540 (0.410–0.670) versus 0.666 (0.555–0.777) at M-12, 0.513 (0.383–0.643) versus 0.679 (0.567–0.791) at M-9, 0.565 (0.431–0.699) versus 0.744 (0.635–0.854) at M-6, 0.670 (0.544–0.796) versus 0.829 (0.740–0.919) at M-3, 0.709 (0.588–0.830) versus 0.821 (0.718–0.925) at M-0. In validation stage, 5.0 ng/ml (Youden Index = 0.667) and 32.0 mAU/ml (Youden Index = 0.509) were calculated as proper cut-off values for following analysis, according to ROC curve.

It has been reported that 100 mAU/ml for PIVKA-II and 200 ng/ml for AFP were decisive cut-off values for HCC detection^16^,^17^. We then calculated the sensitivity and specificity of AFP and PIVKA-II alone and combined at two fixed cut-off values (see Table 2). The closer to the diagnostic time, the more accurate the biomarkers performed. For the combination, only one marker above cut-off value gave a much better sensitivity, but both markers above cut-off value presented poor results.

### Pooled analysis of baseline information

All cases in discovery stage and validation stage at diagnostic time were pooled together for analysis. Categorical variables (gender, age, cirrhosis and BCLC) were compared to figure out if they had impact on the biomarker levels. There was no difference both in AFP and PIVKA-II values (P = 0.689 and 0.280) between male and female. Neither were the impact of age (>40 versus ≤40 years old) and cirrhotic basis, and P values were 0.218 versus 0.094 and 0.673 versus 0.696, respectively. AFP values in early HCC patients and advanced HCC patients (BCLC B+C+D) showed no difference (P = 0.720), but PIVKA-II value elevated significantly in advanced stage compared with early stage (P = 0.005).

**Figure 3:**
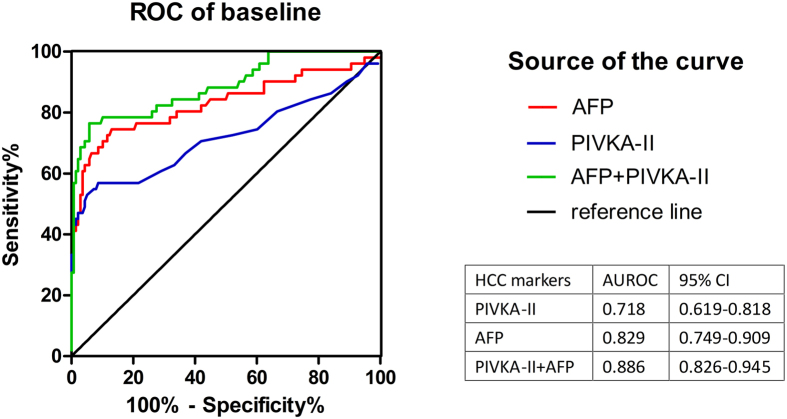
ROC curve at diagnosis for all HCC cases. The AUC is shown with its 95% confidence intervals. The combination of AFP and PIVKA-II increases the diagnostic performance compared with single marker.

Figure 3 shows the ROC curve for AFP, PIVKA-II and the combination of the two biomarkers in diagnosing HCC. Here, we used a new variable log AFP + 4.6*log PIVKA-II to represent the combination of AFP and PIVKA-II^18^. The AUC for PIVKA-II was 0.718 (0.619–0.818) and for AFP was 0.829 (0.749–0.909), while the AUC for the combination was 0.886 (0.826–0.945).

Sensitivities and specificities of AFP and PIVKA-II at different cut-off values were calculated and given on Table 3. PIVKA-II of 32 mAU/ml and AFP of 5.0 ng/ml still gave the best detecting performance compared with other cut-off values, and sensitivity, specificity, Youden index, positive predictive value, negative predictive value, diagnostic accuracy, Kappa index, χ^2^ were 54.9%, 92.8%, 47.7%, 71.8%, 84.7%, 83.3%, 0.520, 40.0 for PIVKA-II and 74.5%, 87.0%, 61.5%, 67.9%, 90.2%, 84.0%, 0.593, 51.0 for AFP, respectively.

### Sequential serum levels from M-12 to diagnosis

**Figure 4:**
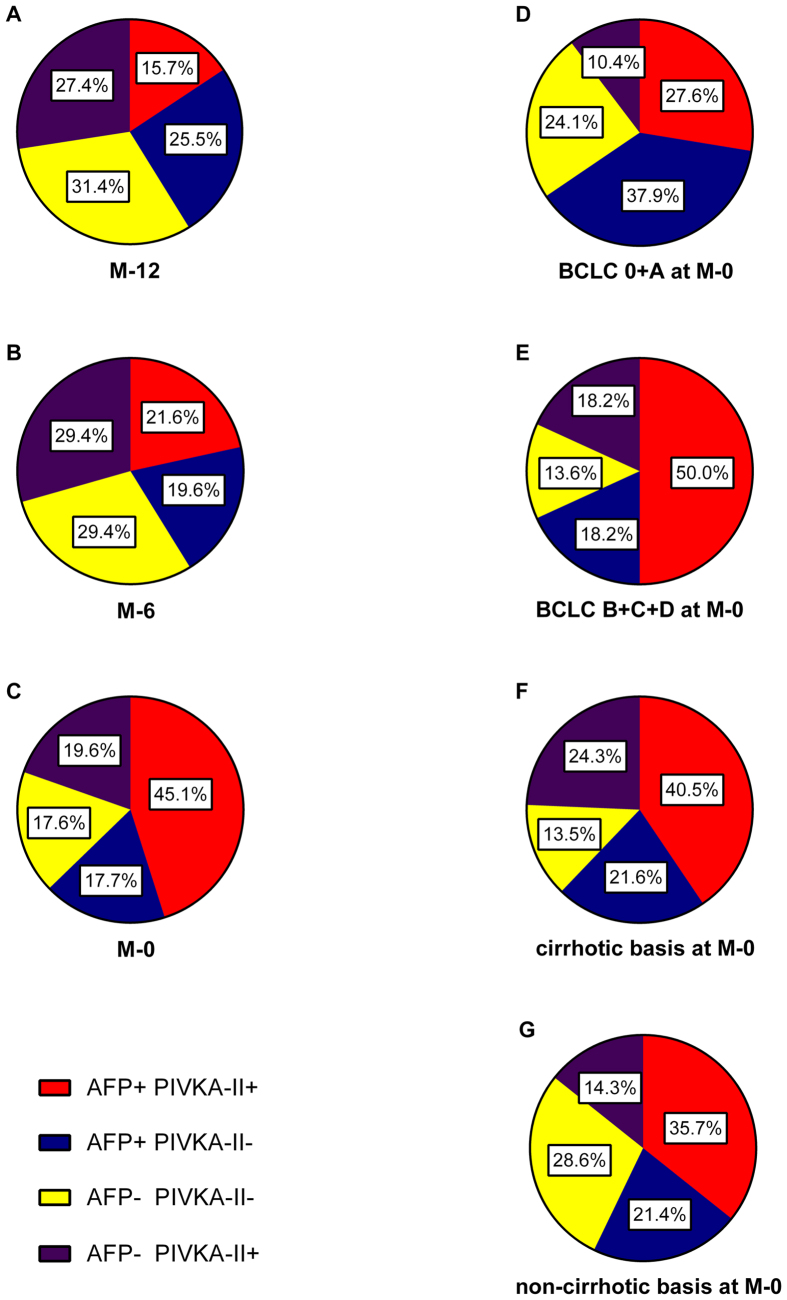
Pie charts of positive rate at all time points for HCC patients. (**A–C**) show the positive rate of every marker in all HCC cases, while (**D,E**) show the positive rate for different stage HCCs and (**F,G**) show the positive rate for different etiological basis of HCC. BCLC, Barcelona Clinic Liver Cancer.

Pie legends shows the change trend of positive ratio of AFP, PIVKA-II for HCC patients at both discovery stage and validation stage (Fig. 4). A, B and C show the change from the time M-12 to diagnosis time in all HCC patients. There were more patients of PIVKA-II+/AFP- than that of PIVKA-II-/AFP+ at all time (27.4% vs 25.5%, 29.4% vs 19.6% and 19.6% vs 17.7%), and patients of PIVKA-II-/AFP- decreased from 31.4% to 17.6% but still took up a large portion. D, E and C show the positive ratio of biomarkers in patients of different HCC stages. In patients of early stage HCC, PIVKA-II-/AFP+ patients were more than PIVKA-II+/AFP- patients (37.9% vs 10.4%), while equal number of PIVKA-II-/AFP+ and PIVKA-II+/AFP- (18.2%) was found if adding stage B and advanced stage patients. F and G show the positive ratio of biomarkers in patients of different etiological basis. Patients of two biomarkers negative were more in HCC patients of non-cirrhotic basis than in cirrhotic HCC patients (28.6% vs 13.5%).

Entirely, levels of the two biomarkers elevated as it came close to the diagnosis time, but from individual aspect, there existed tremendous diversity in all HCC patients (see Fig. 2, Supplemental Content, which illustrates individual serum level of AFP and PIVKA-II in each HCC patients at validation stage). PIVKA-II in some patients (11 out of 36) had great prediction efficacy, whose levels were above cut-off value 12 months prior to diagnosis. But in some patients (7 out of 36), AFP performed better. However, circumstances were complicated in most cases (18 out of 36), the levels were either ups and downs with time goes by or below the cut-off value at all time.

## Discussion

The diagnostic efficiency of PIVKA-II has been illustrated sufficiently and it has been proven that PIVKA-II is a potent biomarker and independent with AFP^19^,^20^,^21^. Nevertheless, most researches concentrate more on diagnosis time when the enhanced CT/MRI or biopsy is golden criterion. But before imaging apparatus could detect abnormality, in the view of cellular level, millions of cells have underwent tumorigenesis, secreting special protein or cytokine. This is why we focus on the predictive efficacy of PIVKA-II before HCC imaging diagnosis.

This nested case-control study included two stage. In discovery stage, levels of both biomarkers were higher in HCC patients than controls. Although in sera of HCC patients, each levels did not increase significantly with time, it is obvious that baseline levels of AFP and PIVKA-II elevated significantly in HCC patients. This increase happened before imaging diagnosis suggesting that AFP and PIVKA-II are proper biomarkers closely linked to HCC tumorigenesis. In other words, both markers are efficient predictors for HCC before imaging discovery. This was verified in validation stage. Compared with controls, levels of markers elevated at all time points in HCC patients. However, in contrast to controls, values in patients were dispersed in a wide range. This, in some aspect, suggested the great heterogeneity among HCC itself.

In validation stage, a detailed comparison was conducted. At every time point, ROC curves for AFP and PIVKA-II were depicted to illustrate predictive and diagnostic performance. At the mention of diagnostic performance, the results vary a lot. Many researches gave optimistic results on PIVKA-II, and they found that PIVKA-II was proper and even better than AFP^22^,^23^,^24^. Whereas, *Marrero JA et al*. and *Grazi GL et al*. admitted that PIVKA-II was poor compared with AFP^25^,^26^. But one thing was unanimous that the combination of two biomarkers was superior to single use^26^,^27^,^28^. Apparently, AUC for AFP was higher than PIVKA-II at each time in our research, and all HCC cases combined gave the same results (0.718 vs 0.829). Positive rate of AFP for HCC cases was 62.8%, but combined with PIVKA-II would increase 19.6% and the AUC increased to 0.886 at diagnosis time. This is consistent with our previous research^29^, indicating that AFP was an irreplaceable biomarker but still insufficient, while PIVKA-II was a favourable complement. Yet, many previous researches had manifested that PIVKA-II represented better than AFP in early stage HCC, but our results seems different. In BCLC 0 and A HCC patients, 37.9% were AFP+/PIVKA-II- and 10.4% were AFP-/PIVKA+ cases. However, no matter at what time and in what stage, combination will substantially increase detection rate.

Actually, neither AFP nor PIVKA-II was enough for HCC detection. This is why guidelines of AASLD and EASL never recommend serological tests as surveillance strategy^6^,^7^. However, the predictive value of serological markers should not be ignored. Although our results showed that AFP was better than PIVKA-II in diagnosis, PIVKA-II seemed better in predicting HCC, which is inconsistent with the conclusion of HCV-based HALT-C trail^14^. At all time-points prior to HCC diagnosis, levels of serological markers had elevated, but there were more patients of PIVKA-II+ alone than AFP+ alone, and 6 months before diagnosis seems the best time for HCC prediction using PIVKA-II with a positive rate of 51.0%. Nevertheless, it should not be denied that patients of AFP+ alone still covered a large part, and this also suggested that the combination of markers was the best method. Individually, serological markers in each HCC patients dramatically varied, but PIVKA-II level in 11 out of 36 patients elevated above cut-off values at all time points prior to diagnosis, contrast with 7 out of 36 for AFP (see Fig. 2, Supplemental Content, which illustrates individual serum level of AFP and PIVKA-II in each HCC patients at validation stage). Consequently, we believed that PIVKA-II is necessary for HCC prediction in HBV-related patients.

At the same time, we cannot neglect a large part of AFP-/PIVKA-II- HCC cases. *Nakao A et al*. reported no AFP-/PIVKA-II- cases^19^ and *Beale G et al*. reported 6.0% cases^30^, but *Carr BI et al*. reported 15.2%^31^ and *Cui R et al*. reported 21.7%^32^. Our data showed that at time M-12, 31.4% were two markers negative and at time M-6 it was 29.4%. Even at diagnosis time, 17.6% cases were still buried but could be found by ultrasound. This demonstrated that ultrasound is helpless when the cancerization happen at cellular level. But secretion of cancer cell has started at that time, so new markers are urgently required to detect the 31.4% or 29.4% cases before imaging diagnosis.

The strength of our research lies in that we collected sequential sera making a cohort. In this cohort, we closely monitor serological changes of each markers so that the differences could be detected. Besides, every patients underwent at least 4-year follow-up, making sure that all data were stable, credible and representative. However, our research is conducted retrospectively, and as a result, the time points are not that precise and few data are missing or unregistered. To avoid this, all cases lack of necessary information were discarded without hesitation. As a result, our HCC cases are less numerous and that is why we separate our research into two stages.

In conclusion, our research proved that both AFP and PIVKA-II had the potential for HCC prediction but patients of PIVKA-II positive were more than AFP positive at the time prior to HCC diagnosis. The combination of two markers could improve the detection rate, but it is not enough. New serological markers or imaging methods should be discovered or developed to discover HCC of all detection targets negative.

## Methods

### Cohort sample source

We used the Hepatitis Biobank of Southwest Hospital (HBS) sample cohort dataset provided by the Department of Infectious Diseases, Southwest Hospital. The HBS dataset corresponded to about 450000 patients chronically infected with HBV in Southwest Hospital since 2001. 3327 patients with more than 4 years follow-up were enrolled. Finally, we screened out patients with hepatitis B surface antigen (HBsAg) negative, with HCV/hepatitis D virus (HDV)/hepatitis E virus (HEV) infection, with liver transplantation history, with other cancer records and with autoimmune hepatitis. In total, 3172 patients with chronic hepatitis B (CHB) were qualified for our research.

Any participants diagnosed as HCC should met two imaging criteria (hepatic ultrasound plus CT or MRI), and then all cases were confirmed by biopsy. Here, early stage HCC was defined as Barcelona Clinic Liver Cancer (BCLC)^33^ classification stage 0 and A. CHB was defined as chronic necroinflammatory liver damage caused by persistent HBV infection. CHB patients also included patients with HBV-induced liver cirrhosis who were confirmed by biopsy. Patients receiving warfarin or vitamin K before haemospasia were screened out for the influence on PIVKA-II level.

### Nested case-control study

This nested case-control study was designed to test if PIVKA-II could detect HCC in advance during a 12-month period before imaging diagnosis. In brief, our study consisted of two stages, discovery stage and validation stage (see Fig. 1, Supplemental Content, which illustrates the selection procedure based on the HBS cohort dataset). We checked all the 3172 cases from our research cohort to screen for HCC cases (a total of 113 HCC cases). Follow-up patients with stored blood samples at the time of HCC diagnosis (M-0) and 12 (M-12), 6 (M-6) months before diagnosis were classified into discovery stage, a total of 15 HCC cases. Correspondingly, at-risk controls were randomly selected and 1:2 matched in age (±1 year), gender and cirrhotic basis. Discovery stage was designed to clarify if liver function had impact on levels of two biomarkers to further provide an unbiased criterion for selecting matches in validation stage.

Likewise, HCC patients repeating ultrasound examinations every 6 months and preserving blood samples at the time of HCC diagnosis and 12, 9 (M-9), 6, 3 (M-3) months before diagnosis were classified into validation stage. Finally, 36 confirmed HCC were qualified. Then for each case, 3 controls were randomly selected from the HBS, which matched in accordance with the results of discovery stage. To avoid subclinical HCC at time 0, only patients with more than 18 months follow-up ahead of time 0 with no clue of HCC were chosen for the matching group.

### Measurement of PIVKA-II and AFP

Serum levels of PIVKA-II were determined by chemiluminescence enzyme immunoassay (CLEIA) (LUMIPULSE® G1200, FUJIREBIO INC., Japan). Serum levels of AFP were measured by AFP Reagent kit via chemiluminescent microparticle immunoassay (CMIA) (ARTHITECT i2000, Abbott Laboratories, America).

### Statistical analysis

All the statistical analyses were performed at SPSS version 18.0 statistical software (IBM, USA) and the graphs were constructed on the Prism version 5.01 (GraphPad Software Inc., USA). Each variable was represented as median with interquartile range. Kolmogorov-Smirnov tests that all variables were skewed data, so Mann-Whitney test was applied to compare the differences between categorical variables (gender, age, cirrhosis and BCLC). Sensitivity, specificity, Kappa value and diagnostic accuracy were calculated by 2 × 2 table in SPSS. Pearson Chi-square test was employed to evaluate statistical differences of diagnostic performance at different cut-off values. In discovery stage, logistic regression was conducted to screen for parameters that have impact on biomarkers. Receiver operating characteristic (ROC) curve was applied for analysing the predicting or diagnostic performance. The area under the curve (AUC) and its 95% confidence interval (CI) were also performed automatically by SPSS. Two-tailed P value less than 0.05 was defined to be statistically significant.

### Ethics statement

The study involved in the manuscript was approved by the ethics committee of Southwest Hospital, Chongqing, China. As a retrospective study, informed consent of research use of surplus blood after clinical laboratory test was obtained from each patient included in the study and the study protocol conforms to the ethical guidelines of the 1975 Declaration of Helsinki as reflected in a priori approval by the institution’s human research committee.

## Additional Information

**How to cite this article**: Yu, R. *et al*. Efficacy of PIVKA-II in prediction and early detection of hepatocellular carcinoma: a nested case-control study in Chinese patients. *Sci. Rep*. **6**, 35050; doi: 10.1038/srep35050 (2016).

## Supplementary Material

Supplementary Information

## Figures and Tables

**Table 1:** Clinical characteristics of HCC cases and controls at baseline point.

	Discovery stage			Validation stage		
	HCC cases	Matched controls	P value	HCC cases	Matched controls	P value
Num. of patients	15	30	..	36	108	..
Age, years	48.0(40.0–66.0)	47.0(39.0–67.0)	0.994	45.5(39.5–52.0)	46.0(39.0–52.0)	0.992
Gender, female	3(20.0%)	6(20.0%)	..	10(27.8%)	30(27.8%)	..
Labs						
TP, g/l	20.1(15.4–24.5)	12.6(10.9–17.2)	0.024	8.1(5.5–30.0)	4.3(2.5–7.0)	0.001
AST, UI/l	28.0(20.0–39.0)	25.0(20.0–32.5)	0.130	30.5(25.0–44.0)	29.0(22.0–37.0)	0.585
ALT, UI/l	37.0(29.0–47.0)	27.0(17.5–39.0)	0.182	31.0(26.0–39.0)	25.0(18.0–39.0)	0.807
ALP, UI/l	74.0(63.0–91.0)	71.0(56.0–94.0)	0.498	90.5(71.0–120.0)	77.0(65.0–92.0)	0.018
TBIL, μmol/l	78.4(74.9–80.0)	77.6(74.2–80.2)	0.962	76.5(70.5–79.7)	79.3(74.9–82.3)	<0.001
TBA, μmol/l	4.3(3.3–9.2)	4.1(2.4–7.3)	0.895	18.9(12.0–25.7)	13.6(10.5–19.1)	0.002
HBV DNA (log10 copies/ml)						
Undetectable*	10(67.6%)	22(73.3%)	..	29(80.5%)	86(79.6%)	..
2.69–3.99	3(20.0%)	5(16.7%)	..	5(13.9%)	13(12.1%)	..
4.00–5.99	1(6.7%)	1(3.3%)	..	2(5.6%)	5(4.6%)	..
≥6.00	1(6.7%)	2(6.7%)	..	0(0)	4(3.7%)	..
HCC markers						
AFP, ng/ml	28.9(4.3–160.5)	2.9(2.3–5.5)	<0.001	10.7(3.6–130.4)	3.1(2.2–3.6)	<0.001
Log_10_AFP, ng/ml	1.5(0.6–2.2)	0.5(0.4–0.7)	<0.001	1.0(0.6–2.1)	0.5(0.3–0.6)	<0.001
PIVKA–II, mAU/ml	31.0(22.0–253.0)	20.0(18.0–25.0)	0.010	34.0(19.0–437.5)	21.0(18.0–26.0)	0.033
Log_10_PIVKA-II, mAU/ml	1.5(1.3–2.4)	1.3(1.2–1.4)	0.001	1.5(1.3–2.6)	1.3(1.3–1.4)	<0.001
Cirrhosis	10(67.7%)	20(67.7%)	..	27(75.0%)	81(75.0%)	..
BCLC stage						
0+A	11(73.3%)	..	..	18(50.0%)		..
B	3(20.0%)	..	..	14(38.9%)		..
C+D	1(6.7%)	..	..	4(11.1%)		..

Data are presented as n (%), or median with IQR (the 25th and the 75th percentiles).

HCC: hepatocellular carcinoma; TP: total protein; AST: aspartate aminotransferase; ALT: alanine aminotransferase; ALP: alkaline phosphatase; TBIL: total bilirubin; TBA: total bile acid; HBV: hepatitis B virus; BCLC: Barcelona Clinic Liver Cancer.

*Copies under 500 are clinically undetectable.

**Table 2:** Sensitivity and Specificity of PIVKA-II and AFP in differentiating HCC cases from controls at two fixed cut-off values.

Months before HCC diagnosis	Sensitivity	Specificity	Youden Index	Sensitivity	Specificity	Youden Index
PIVKA-II (mAU/ml)	≥32.0			≥100		
−12	31.4%	93.5%	24.9%	2.9%	100%	2.9%
−9	27.8%	94.4%	22.2%	5.6%	99.1%	4.7%
−6	38.9%	92.6%	31.5%	8.3%	100%	8.3%
−3	41.7%	92.6%	34.3%	19.4%	100%	19.4%
0	58.3%	92.6%	50.9%	36.1%	100%	36.1%
AFP (ng/ml)	≥5.0			≥200		
−12	51.4%	77.8%	29.2%	2.9%	100%	2.9%
−9	47.2%	77.8%	25.0%	0	100%	0
−6	55.6%	88.0%	43.6%	5.6%	100%	5.6%
−3	66.7%	88.9%	55.6%	16.7%	100%	16.7%
0	75.0%	91.7%	66.7%	19.4%	100%	19.4%
AFP +PIVKA-II	≥5.0 or * ≥32.0			≥5.0 and ** ≥32.0		
−12	27.8%	75.9%	3.7%	13.8%	98.1%	11.9%
−9	61.1%	75.0%	36.1%	13.8%	98.1%	11.9%
−6	72.2%	82.4%	54.6%	22.2%	99.1%	21.3%
−3	77.8%	82.4%	60.2%	16.7%	100%	16.7%
0	88.9%	85.2%	74.1%	25.0%	100%	25.0%

PIVKA-II: Protein Induced by Vitamin K Absence or Antagonist-II; AFP: α-fetoprotein; HCC: hepatocellular carcinoma.

*or: Positive is defined as either PIVKA-II or AFP above cut-off value.

**and: Positive is defined as both PIVKA-II and AFP above cut-off value.

**Table 3:** Performance of PIVKA-II and AFP in differentiating HCC cases from controls at different cut-off values at diagnostic time.

Cut-off values	Sensitivity	Specificity	Youden Index	Diagnostic accuracy	Positive predictive value	Negative predictive value	Kappa index	χ^2^
PIVKA-II (mAU/ml)								
32	54.9%	92.8%	47.7%	83.3%	71.8%	84.7%	0.520	40.0
40	45.1%	97.8%	42.9%	83.6%	88.5%	82.8%	0.508	57.8
80	37.3%	99.3%	36.6%	82.5%	95.0%	81.1%	0.452	52.5
400	23.5%	100%	23.5%	79.4%	100%	78.0%	0.310	34.7
AFP (ng/ml)								
5	74.5%	87.0%	61.5%	84.0%	67.9%	90.2%	0.593	51.0
20	43.1%	98.6%	41.7%	83.6%	91.7%	82.4%	0.500	58.4
40	35.3%	99.3%	34.6%	82.0%	94.7%	80.6%	0.431	49.2
200	19.6%	100%	19.6%	78.3%	100%	77.1%	0.263	28.6

PIVKA-II: Protein Induced by Vitamin K Absence or Antagonist-II; AFP: α-fetoprotein; HCC: hepatocellular carcinoma.
