# Corrigendum: Efficacy of PIVKA-II in prediction and early detection of hepatocellular carcinoma: a nested case-control study in Chinese patients

**DOI:** 10.1038/srep39184

**Published:** 2016-12-19

**Authors:** Rentao Yu, Xiaomei Xiang, Zhaoxia Tan, Yi Zhou, Haoliang Wang, Guohong Deng

Scientific Reports 6: Article number: 3505010.1038/srep35050; published online: 10 12 2016; updated: 12 19 2016

This Article contains errors in Table 2, where the ‘Sensitivity’ and ‘Youden Index’ values for AFP + PIVKA-II (≥5.0 or ≥32.0) are incorrectly given as 27.8% and 3.7% respectively. The correct Table 2 appears below.

## Figures and Tables

**Table 2:** Sensitivity and Specificity of PIVKA-II and AFP in differentiating HCC cases from controls at two fixed cut-off values.

Months before HCC diagnosis	Sensitivity	Specificity	Youden Index	Sensitivity	Specificity	Youden Index
PIVKA-II (mAU/ml)	≥32.0			≥100		
−12	31.4%	93.5%	24.9%	2.9%	100%	2.9%
−9	27.8%	94.4%	22.2%	5.6%	99.1%	4.7%
−6	38.9%	92.6%	31.5%	8.3%	100%	8.3%
−3	41.7%	92.6%	34.3%	19.4%	100%	19.4%
0	58.3%	92.6%	50.9%	36.1%	100%	36.1%
AFP (ng/ml)	≥5.0			≥200		
−12	51.4%	77.8%	29.2%	2.9%	100%	2.9%
−9	47.2%	77.8%	25.0%	0	100%	0
−6	55.6%	88.0%	43.6%	5.6%	100%	5.6%
−3	66.7%	88.9%	55.6%	16.7%	100%	16.7%
0	75.0%	91.7%	66.7%	19.4%	100%	19.4%
AFP +PIVKA-II	≥5.0 or ≥32.0			≥5.0 and ≥32.0		
−12	77.2%	75.9%	53.1%	13.8%	98.1%	11.9%
−9	61.1%	75.0%	36.1%	13.8%	98.1%	11.9%
−6	72.2%	82.4%	54.6%	22.2%	99.1%	21.3%
−3	77.8%	82.4%	60.2%	16.7%	100%	16.7%
0	88.9%	85.2%	74.1%	25.0%	100%	25.0%

PIVKA-II: Protein Induced by Vitamin K Absence or Antagonist-II; AFP: α-fetoprotein; HCC: hepatocellular carcinoma.

*or: Positive is defined as either PIVKA-II or AFP above cut-off value.

**and: Positive is defined as both PIVKA-II and AFP above cut-off value.

