# Supplementary material for: Efficacy of PIVKA-II in prediction and early detection of hepatocellular carcinoma: a nested case-control study in Chinese patients
Source: Sci Rep. 2016 Oct 12;6:35050. doi: 10.1038/srep35050 (PMC5059731; doi:10.1038/srep35050)
Supplement: Supplementary Information [file srep35050-s1.pdf]

Supplementary materials to:

Efficacy of PIVKA-II in prediction and early detection of hepatocellular carcinoma: a nested case-control study in Chinese patients

Yu Rentao[1,2], Xiang Xiaomei[1,2], Tan Zhaoxia[1,2], Zhou Yi[1,2], Wang Haoliang[3], Deng Guohong[1,2,3]

[1] Department of Infectious Diseases, Southwest Hospital, the Third Military Medical University, Chongqing, China

[2] Chongqing Key Laboratory of Infectious Diseases, Southwest Hospital, Third Military Medical University, Chongqing, China

[3] Institute of Immunology, Third Military Medical University, Chongqing, China

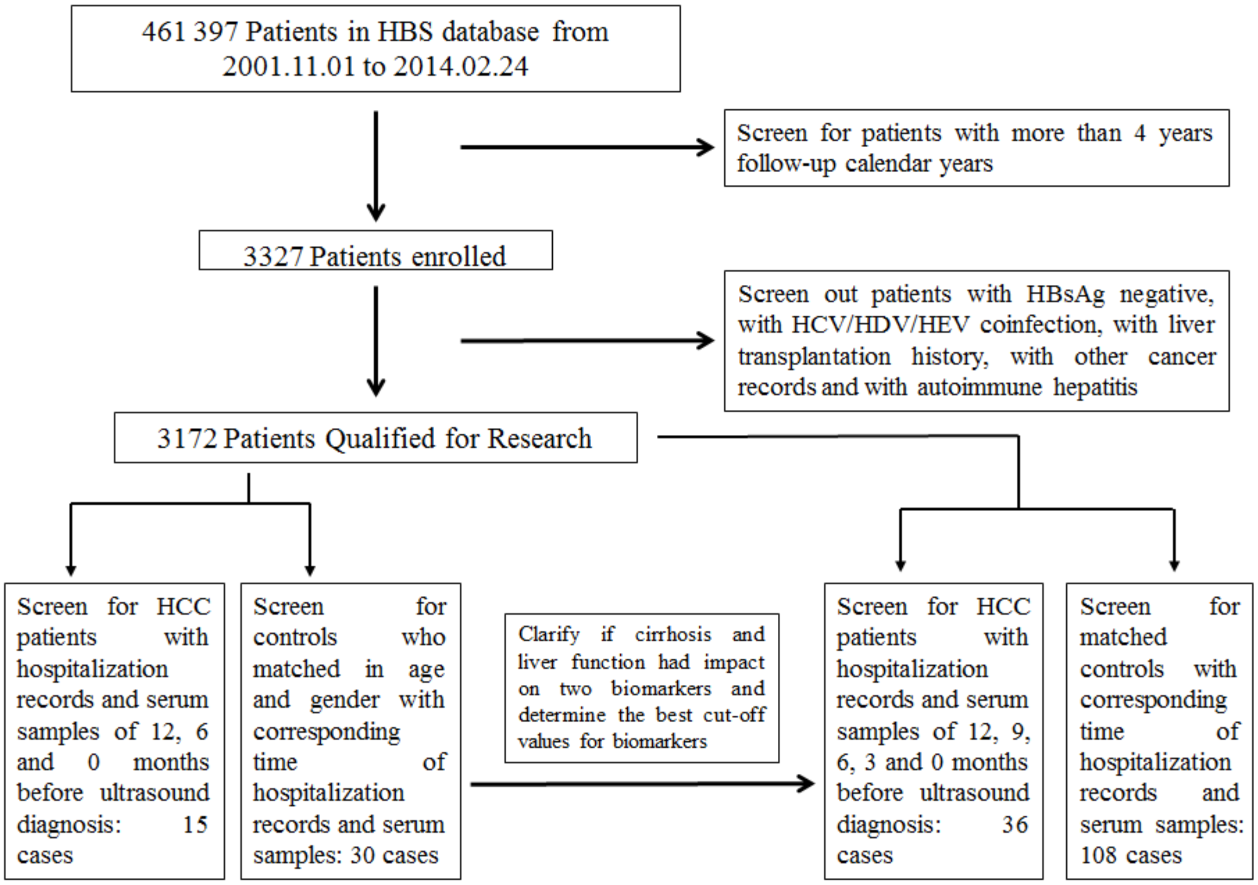

Supplementary Fig. 1 Flow chart representing the selection procedure based on the HBS cohort dataset. All HCC patients were checked for hospitalization records and sera samples and patients with regular follow-up records were selected for use. Totally, 51 cases (most were early-stage HCC) were qualified. In the end, 15 cases with -12, -6 and 0 months before diagnosis were classified into discovery stage and for each case, 2 controls were selected from the same cohort dataset for matching. So did the validation stage, but there were 1:3 matched controls in validation stage.

Supplementary Table 1 Multivariable analysis of HCC in discovery stage.

	AFP	PIVKA	gender	age	cirrhosis	AST	ALT	ALP	TBIL	TBA	TP	lgDNA
P	0.019	0.022	0.921	0.261	0.473	0.212	0.296	0.142	0.518	0.632	0.12	0.398
OR	11.086	11.023	0.002	0.912	7.528	0.808	1.327	0.928	1.1	0.93	2.292	0.522
95% CI	0.893	0.986	4.77E-57	0.776	3.55	0.578	0.78	0.84	0.824	0.692	0.805	0.116
	1.321	1.062	8.28E+50	1.071	13.845	1.13	2.258	1.025	1.468	1.25	6.526	2.358

In this binary logistic regression, the state of HCC was set as dependent variable, and biomarkers, cirrhosis basis and bio-parameters reflecting liver function were set as covariates. AFP and PIVKA-II were risk factors for HCC and P values were below 0.05. OR, odds ratio; CI, confidential interval.

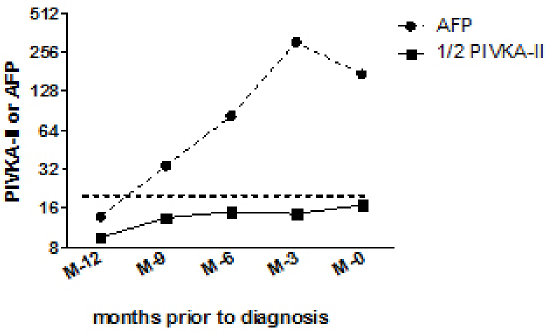

PIVKA-II or AFP
512
256
128
64
32
16
8
M-12
M-9
M-6
M-3
M-0
months prior to diagnosis
AFP
1/2 PIVKA-II

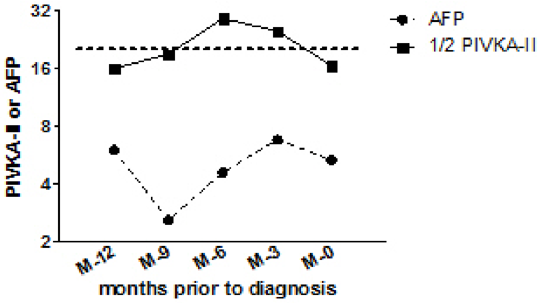

PIVKA-II or AFP
32
16
8
4
2
M-12
M-9
M-6
M-3
M-0
months prior to diagnosis
AFP
1/2 PIVKA-II

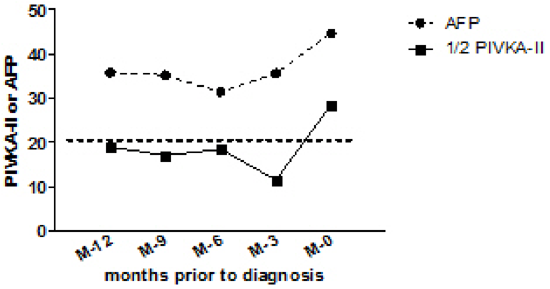

PIVKA-II or AFP
50
40
30
20
10
0
M-12
M-9
M-6
M-3
M-0
months prior to diagnosis
AFP
1/2 PIVKA-II

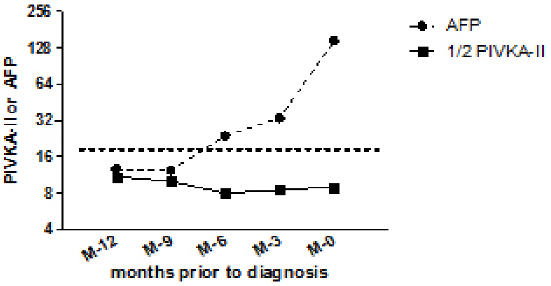

PIVKA-II or AFP
256
128
64
32
16
8
4
M-12
M-9
M-6
M-3
M-0
months prior to diagnosis
AFP
1/2 PIVKA-II

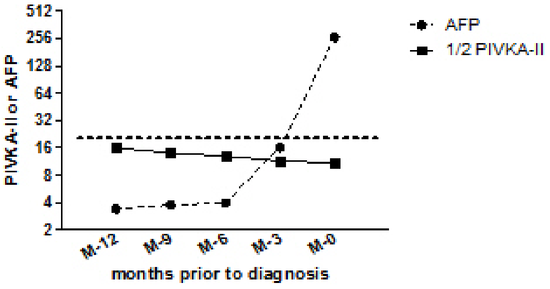

PIVKA-II or AFP
512
256
128
64
32
16
8
4
2
M-12
M-9
M-6
M-3
M-0
months prior to diagnosis
AFP
1/2 PIVKA-II

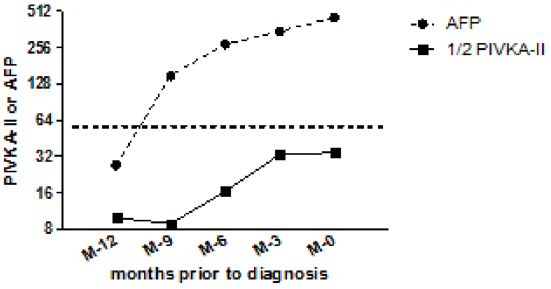

PIVKA-II or AFP
512
256
128
64
32
16
8
M-12
M-9
M-6
M-3
M-0
months prior to diagnosis
AFP
1/2 PIVKA-II

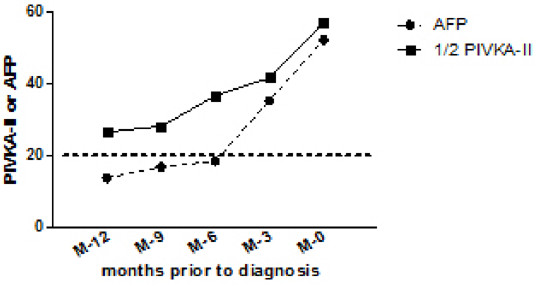
PIVKA-II or AFP
60
40
20
0
M-12
M-9
M-6
M-3
M-0
months prior to diagnosis
AFP
1/2 PIVKA-II

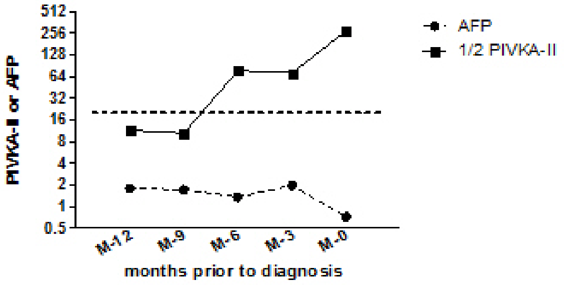
PIVKA-II or AFP
512
256
128
64
32
16
8
4
2
1
0.5
M-12
M-9
M-6
M-3
M-0
months prior to diagnosis
AFP
1/2 PIVKA-II

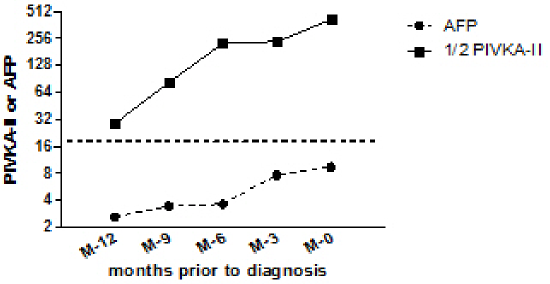
PIVKA-II or AFP
512
256
128
64
32
16
8
4
2
M-12
M-9
M-6
M-3
M-0
months prior to diagnosis
AFP
1/2 PIVKA-II

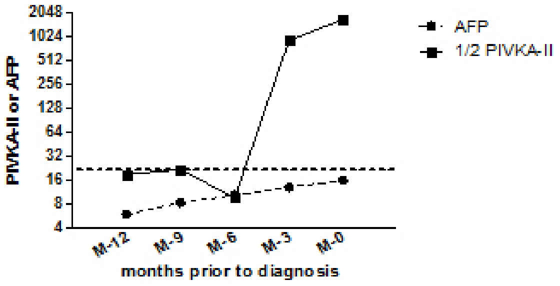
PIVKA-II or AFP
2048
1024
512
256
128
64
32
16
8
4
M-12
M-9
M-6
M-3
M-0
months prior to diagnosis
AFP
1/2 PIVKA-II

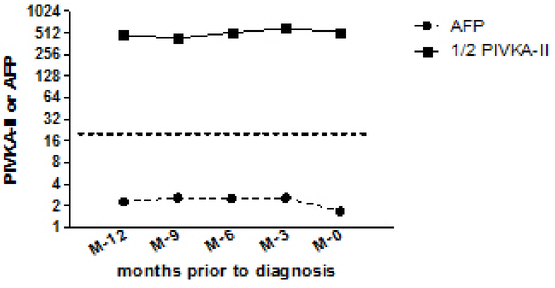
PIVKA-II or AFP
1024
512
256
128
64
32
16
8
4
2
1
M-12
M-9
M-6
M-3
M-0
months prior to diagnosis
AFP
1/2 PIVKA-II

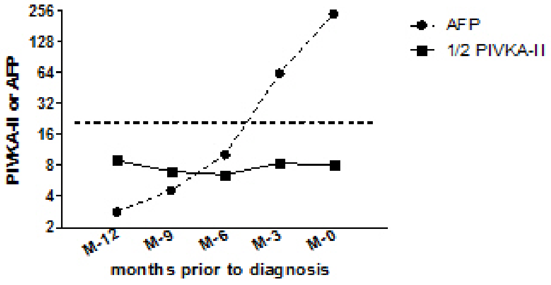
PIVKA-II or AFP
256
128
64
32
16
8
4
2
M-12
M-9
M-6
M-3
M-0
months prior to diagnosis
AFP
1/2 PIVKA-II

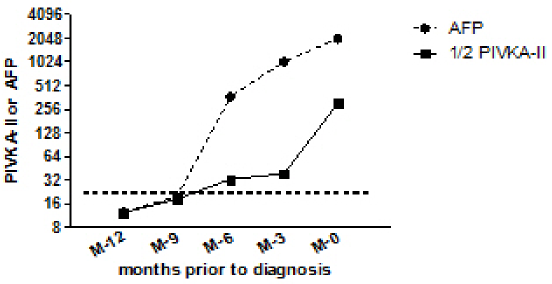
PIVKA-II or AFP
4096
2048
1024
512
256
128
64
32
16
8
M-12
M-9
M-6
M-3
M-0
months prior to diagnosis
AFP
1/2 PIVKA-II

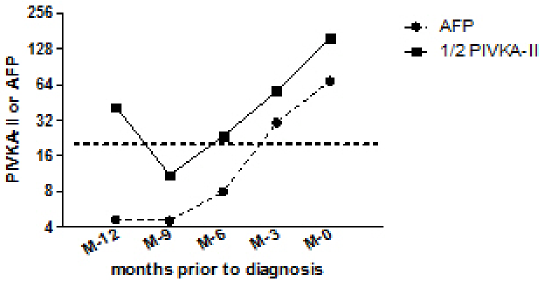
PIVKA-II or AFP
256
128
64
32
16
8
4
M-12
M-9
M-6
M-3
M-0
months prior to diagnosis
AFP
1/2 PIVKA-II

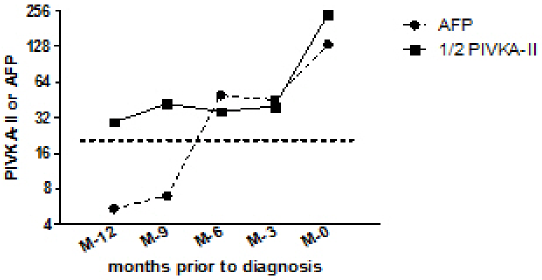
PIVKA-II or AFP
256
128
64
32
16
8
4
M-12
M-9
M-6
M-3
M-0
months prior to diagnosis
AFP
1/2 PIVKA-II

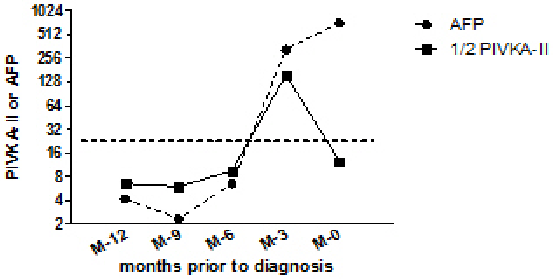
PIVKA-II or AFP
1024
512
256
128
64
32
16
8
4
2
M-12
M-9
M-6
M-3
M-0
months prior to diagnosis
AFP
1/2 PIVKA-II

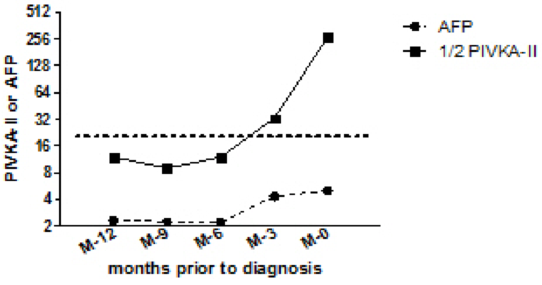
PIVKA-II or AFP
512
256
128
64
32
16
8
4
2
M-12
M-9
M-6
M-3
M-0
months prior to diagnosis
AFP
1/2 PIVKA-II

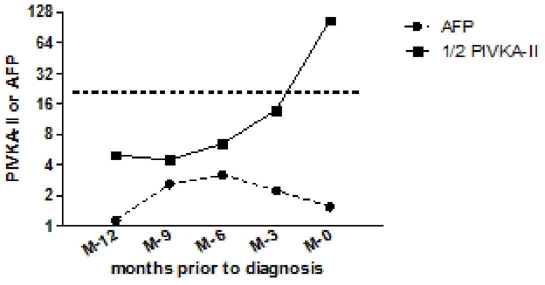
PIVKA-II or AFP
128
64
32
16
8
4
2
1
M-12
M-9
M-6
M-3
M-0
months prior to diagnosis
AFP
1/2 PIVKA-II

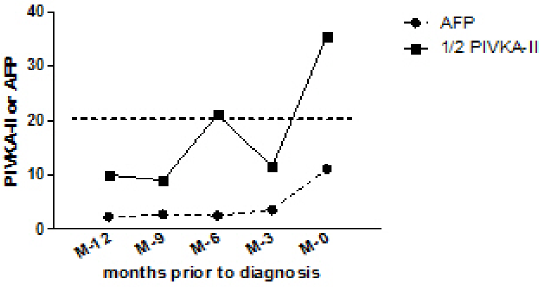

PIVKA-II or AFP
40
30
20
10
0
M-12
M-9
M-6
M-3
M-0
months prior to diagnosis
AFP
1/2 PIVKA-II

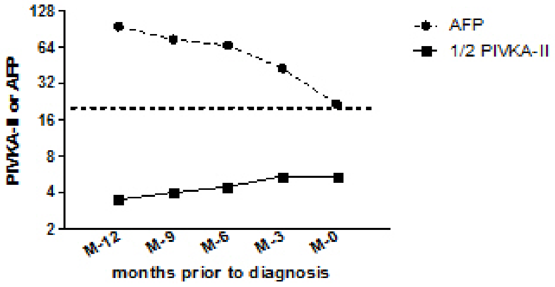

PIVKA-II or AFP
128
64
32
16
8
4
2
M-12
M-9
M-6
M-3
M-0
months prior to diagnosis
AFP
1/2 PIVKA-II

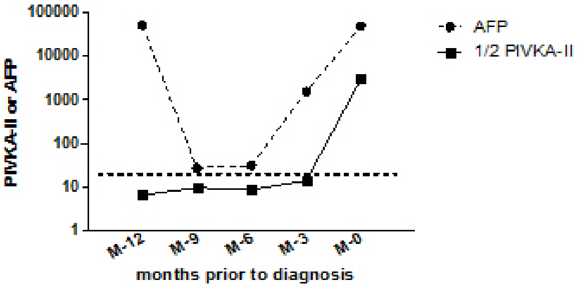

PIVKA-II or AFP
100000
10000
1000
100
10
1
M-12
M-9
M-6
M-3
M-0
months prior to diagnosis
AFP
1/2 PIVKA-II

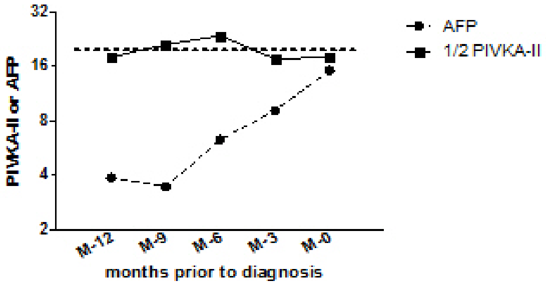

PIVKA-II or AFP
32
16
8
4
2
M-12
M-9
M-6
M-3
M-0
months prior to diagnosis
AFP
1/2 PIVKA-II

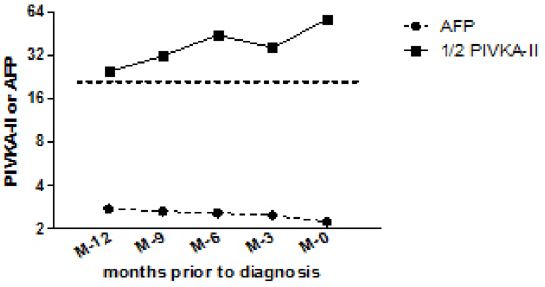

PIVKA-II or AFP
64
32
16
8
4
2
M-12
M-9
M-6
M-3
M-0
months prior to diagnosis
AFP
1/2 PIVKA-II

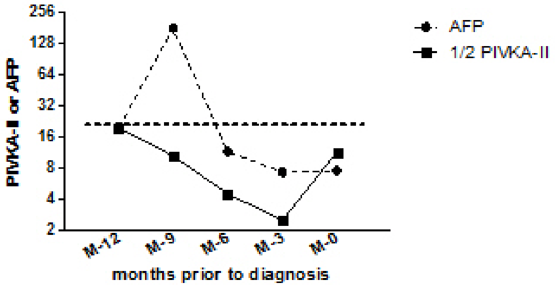

PIVKA-II or AFP
256
128
64
32
16
8
4
2
M-12
M-9
M-6
M-3
M-0
months prior to diagnosis
AFP
1/2 PIVKA-II

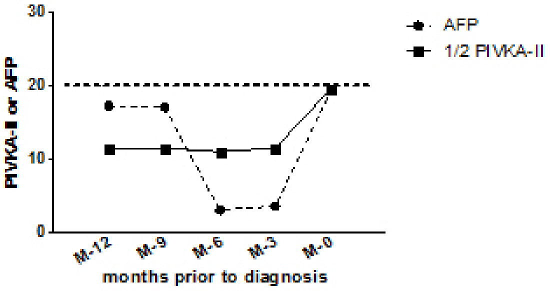
30
20
10
0
PIVKA-II or AFP
M-12
M-9
M-6
M-3
M-0
months prior to diagnosis
AFP
1/2 PIVKA-II

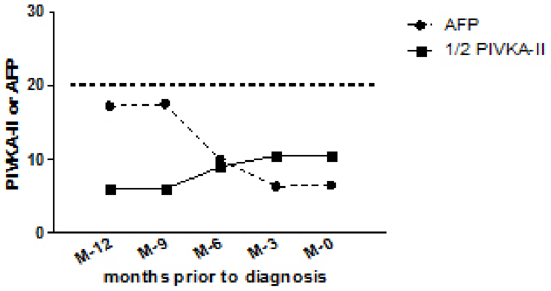
30
20
10
0
PIVKA-II or AFP
M-12
M-9
M-6
M-3
M-0
months prior to diagnosis
AFP
1/2 PIVKA-II

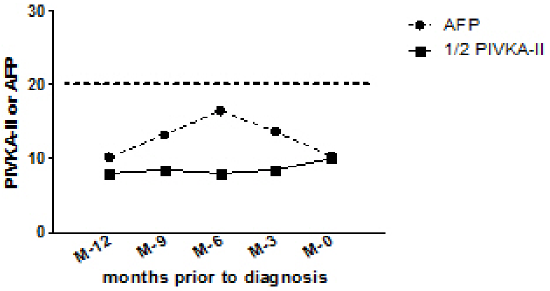
30
20
10
0
PIVKA-II or AFP
M-12
M-9
M-6
M-3
M-0
months prior to diagnosis
AFP
1/2 PIVKA-II

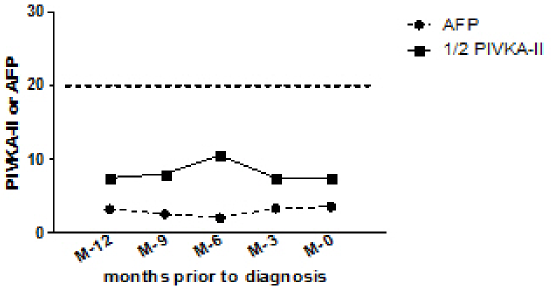
30
20
10
0
PIVKA-II or AFP
M-12
M-9
M-6
M-3
M-0
months prior to diagnosis
AFP
1/2 PIVKA-II

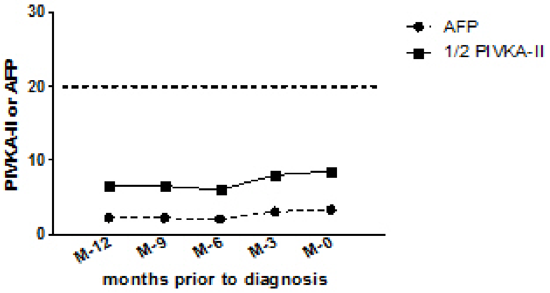
30
20
10
0
PIVKA-II or AFP
M-12
M-9
M-6
M-3
M-0
months prior to diagnosis
AFP
1/2 PIVKA-II

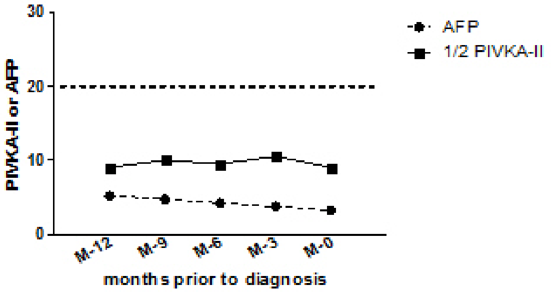
30
20
10
0
PIVKA-II or AFP
M-12
M-9
M-6
M-3
M-0
months prior to diagnosis
AFP
1/2 PIVKA-II

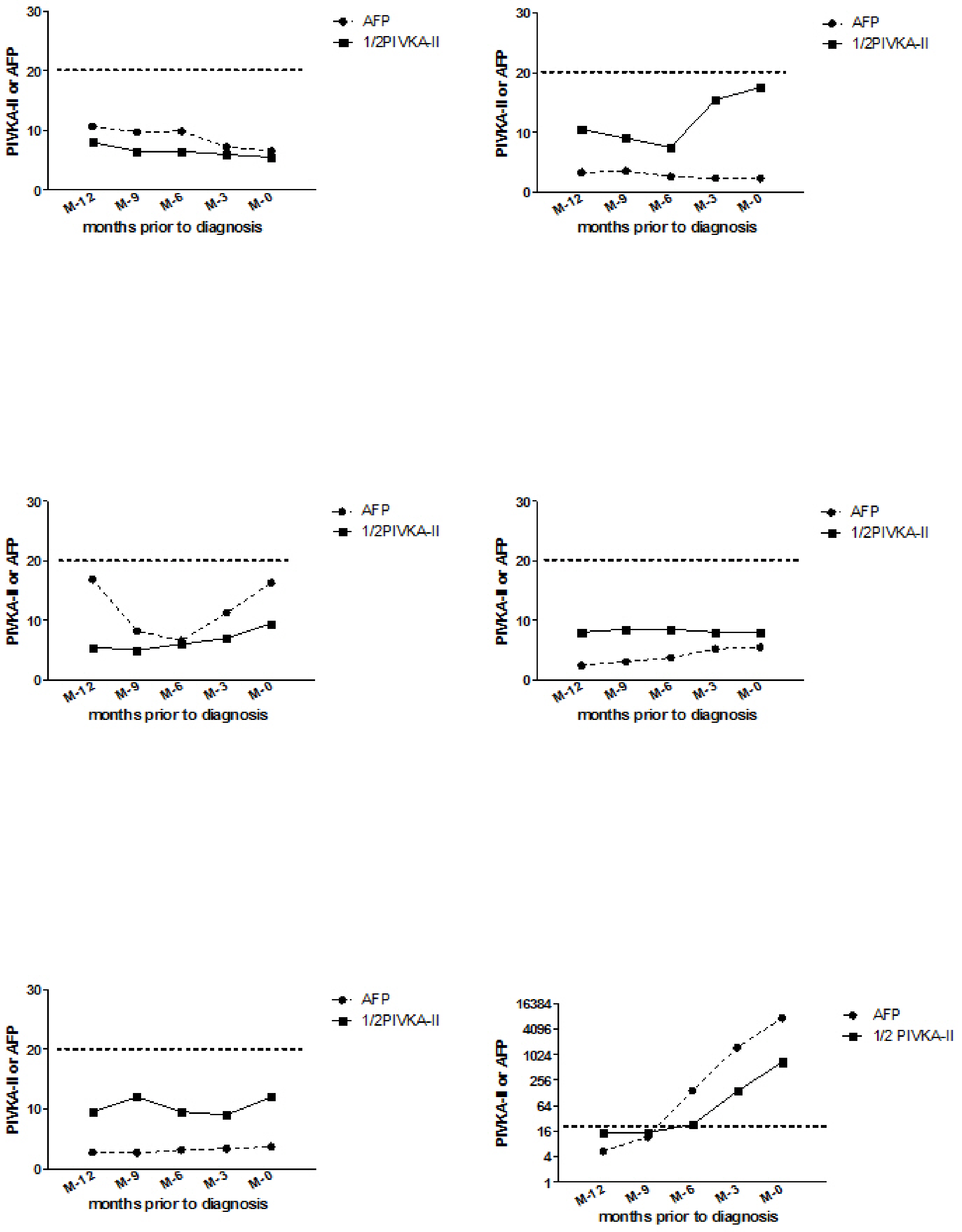

Supplementary Fig. 2 Chang trends of AFP and PIVKA-II serum level in each HCC patients at validation stage. Each figure shows the change trend of biomarkers' trend of a HCC patient from 12 months prior to diagnosis to the diagnosis time. The horizontal dotted line refers to the cut-off value. PIVKA-II levels were reduced to 1/2 so as to keep a same cut-off value as AFP. PIVKA-II: Protein Induced by Vitamin K Absence or Antagonist-II; AFP: α-fetoprotein.
